# Butyrate and obesity: Current research status and future prospect

**DOI:** 10.3389/fendo.2023.1098881

**Published:** 2023-02-24

**Authors:** Ke Peng, Wenjie Dong, Taimin Luo, Hui Tang, Wanlong Zhu, Yilan Huang, Xuping Yang

**Affiliations:** ^1^ Department of Pharmacy, The Affiliated Hospital of Southwest Medical University, Luzhou, China; ^2^ School of Pharmacy, Southwest Medical University, Luzhou, China; ^3^ Department of Pharmacy, Chengdu Seventh People’s Hospital, Chengdu, Sichuan, China

**Keywords:** butyrate, SCFAs, gut microbiota, obesity, metabolic disease

## Abstract

Over the past few decades, increasing prevalence of obesity caused an enormous medical, social, and economic burden. As the sixth most important risk factor contributing to the overall burden of disease worldwide, obesity not only directly harms the human body, but also leads to many chronic diseases such as diabetes, cardiovascular diseases (CVD), nonalcoholic fatty liver disease (NAFLD), and mental illness. Weight loss is still one of the most effective strategies against obesity and related disorders. Recently, the link between intestinal microflora and metabolic health has been constantly established. Butyrate, a four-carbon short-chain fatty acid, is a major metabolite of the gut microbiota that has many beneficial effects on metabolic health. The anti-obesity activity of butyrate has been demonstrated, but its mechanisms of action have not been fully described. This review summarizes current knowledge of butyrate, including its production, absorption, distribution, metabolism, and the effect and mechanisms involved in weight loss and obesity-related diseases. The aim was to contribute to and advance our understanding of butyrate and its role in obesity. Further exploration of butyrate and its pathway may help to identify new anti-obesity.

## 1 Introduction

Over the past few decades, obesity and related health problems have increased with the accessibility to high calorie foods with low nutritional value and changes in lifestyle (1, 2). In the early 2000s, 1.1 billion adults and 10% of children were classified as overweight or obese (3). According to the last data from the World Health Organization, the number of overweight or obese people has risen to more than 1.9 billion adults aged 18 years and older and over 18% of children (4). Obesity is measured by the body mass index (BMI), which is body weight/height squared (kg/m^2^), and people with a BMI ≥30 kg/m^2^ are classified as obese (5). Obesity has been officially recognized as a disease since 1985, and ongoing research has shown that obesity is a serious threat to human health (6). Current evidence confirms that obesity is associated not only with chronic illnesses such as diabetes, atherosclerosis, and cardiovascular disease (CVD) (7–9), but also with cancer (10) and cognitive impairment (11). Beyond this, obesity also severely decreases both physical and mental aspects of the quality of life for people (12, 13). Thus, we need to focus more on the health risks and therapeutic approaches to obesity.

Abnormal or excess accumulation of fat that leads to obesity and impairs health (4), results from an energy imbalance, with more calories consumed than expended (14), and most interventions to control or treat obesity involve calorie restriction (15). Evidence provided by an increasing number of confirms that the composition of the gut microbiota influences the energy metabolism and metabolic health of the human host (16). Changes in human gut microbiota have been linked to obesity, and weight loss influences the composition of the gut microbiota (17). More importantly, supplementation with *Akkermansia muciniphila* (a kind of intestinal microflora) in overweight and obese human volunteers was able to improve obesity or obesity-related phenotypes (18). Short-chain fatty acids (SCFAs), which constitute the most abundant metabolites of microbial fermentation from undigested dietary carbohydrates, are crucial mediators between microbiota and host metabolism (19). The most abundant SCFAs in the human body include acetate (C2), propionate (C3) and butyrate (C4) and they are also the most abundant anions in the colon (20). Among SCFAs, acetate shows more obesogenic effect. Research has shown that chronic increase in acetate would promote chronic hyperinsulinemia, hyperphagia, and weight gain and the associated sequelae of obesity by the activation of the parasympathetic nervous system (21). Acetate also contributes to the synthesis of cholesterol and the synthesis of lipids in liver (22, 23). Recently, evidence indicate that local acetate inhibits brown fat function (24). Propionate has been relatively poorly studied in relation to obesity, although metabolic benefits have also been reported. In SCFA, butyrate has the most important systemic effects. There are a large number of reports that butyrate is inextricably related to obesity and weight loss. Butyrate is known to have beneficial effects on cellular energy metabolism and intestinal homeostasis (25), and to mediate regulation of whole-body energy homeostasis by the gut microbiota (26). Evidence points to the involvement of decreased butyrate production in metabolic diseases such as diabetes (27). Studies of microbiota composition have revealed that diabetic and obese patients have lower levels of butyrate-producing bacteria, while dietary supplementation with butyrate ameliorates inflammation, insulin resistance and weight gain (28, 29). However, the amount of butyrate in stools has been found to be higher in overweight and obese volunteers than in lean volunteers (30, 31), which led some researchers to believe that butyrate may contribute to the obesogenic phenotype (32). However, fecal concentrations may not accurately reflect physiological concentrations since <10% of butyrate production is excreted in feces and mice studies suggested that the obese microbiota actually has a reduced capacity to produce butyrate (33). The specific effect of butyrate on obesity remains unclear. This review aims to contribute to further related research in the future by summarizing current knowledge about the butyrate, especially its effects and mechanisms of action in obesity.

## 2 *In vivo* process of butyrate

### 2.1 Production source of butyrate

Some studies showed that dietary fiber can exert similar effects with butyrate *via* enhanced gut butyrate production (34–36). Thus, an understanding of how butyrate is produced in the gut can help to better understand its effects. It is well known that butyrate is produced from carbohydrate polymers *via* glycolysis by gut microbiota, such as *Eubacterium rectale*, *Faecalibacterium prausnitzii* (37, 38). The fermentation substrates are dietary fibers that are transported to the colon after escaping digestion in the upper gastrointestinal tract (38), and are also commonly known as a kind of dietary fibers including undigested plant polysaccharides, resistant starch (RS), and nondigestible oligosaccharides (NDOs) (39, 40). The undigested plant polysaccharides are usually classified into two types: water-soluble polysaccharides and water-insoluble polysaccharides (38). Water-insoluble fibers such as lignin, cellulose, and some hemicelluloses are resistant to fermentation (41). Water-insoluble polysaccharides are usually hydrolyzed to smaller soluble fragments, and are then fermented to produce butyrate (42). Water-soluble polysaccharides, such as pectin,β-glucans, FOS, inulin and gums, tend to be more completely fermented by colonic microflora (41). Most fiber-containing foods contain about one-third soluble and two-thirds insoluble fiber (43). Resistant starch that was able to resist the digestion in the small intestine will arrive at the colon where they will be fermented by the gut microbiota, producing a variety of products including butyrate (44). RS is usually present in cereal grains, seeds, cooked starch, and starch-containing foods like potato, barley, wheat and corn (44, 45). The nondigestible oligosaccharides (NDOs) are typically saccharides containing between 3 and 10 sugar moieties, including isomalt-oligosaccharides (IMOs), galacto-oligosaccharides (GOSs), XOSs, and some pseudo-oligosaccharides, such as acarbose (38, 46).

Butyrate is also formed as products from peptide and amino acid fermentation, although amino acid-fermenting bacteria have been estimated to constitute less than 1% of the large intestinal microbiota (47). The amino acid which can produce butyrate from microbial fermentation include glutamate, lysine, histidine, cysteine, serine and methionine (48). Apart from that, butyrate also occurs naturally in dairy products, like whole cow’s milk (~0.1 g/100g), butter (~3 g/100 g), cheese (especially goat’s cheese (~1-1.8 g/100 g), parmesan (~1.5 g/100 g) (49), and human breast milk(uptake of an estimated amount of approximately 30mg/kg in a breast-fed baby) (50).

### 2.2 Gut microbiota and pathway of butyrate production

Previously described butyrate-produced microbiota in the human gastrointestinal intestinal tract were commonly distributed in the *phylum Firmicutes* and the *order Clostridiales* (38). Most of these producing bacteria are in four main families: *Clostridiaceae, Eubacteriaceae, Lachnospiraceae*, and *Ruminococcaceae* (37, 51, 52). Most butyrate-produced microbiota in the *order Clostridiales* are widely distributed in several clusters, including clusters IV, XIVa, XVI, and I (53). Moreover, other typical butyrogenic species like *Roseburia spp, Anaerostipes spp, Clostridium spp, Ruminococcus spp, Coprococcus spp* and *Butyrivibrio spp* are widely distributed across cluster XIVa, and *Butyricicoccus pullicaecorum, Subdoligranulum variabile, Anaerotruncus colihominis*, and *Papillibacter cinnamivorans* are cluster IV (51, 52, 54).

Butyrate is produced from carbohydrates by glycolysis that involves the combination of two molecules of acetyl-CoA to form acetoacetyl-CoA followed by stepwise reduction to butyryl-CoA (37). In addition to acetyl-CoA, there are three other pathways known to produce butyrate, glutarate, 4-aminobutyrate, and lysine and all pathways coalesce at a central energy-generating step where crotonyl-CoA is transformed into butyryl-CoA, catalyzed by the electron-transferring flavoprotein butyryl-CoA dehydrogenase (52). Two different pathways are known for the final step in butyrate formation from butyryl-CoA, which proceeds either *via* butyryl-CoA: acetate CoA-transferase or *via* phosphotransbutyrylase and butyrate kinase (51). A screen of 38 butyrate-producing human gut isolates found that the butyryl-CoA: acetate CoA-transferase route was far more prevalent in this ecosystem than the butyrate kinase route (55).

### 2.3 Factors affecting butyrate production

At first, the solubility of fermentation substrate significantly affects the fermentation of NDCs (38). Soluble NDCs are generally more susceptible to fermentation by gut microbiota than insoluble NDCs (56), and highly fermentable to be rapidly consumed by microbes (57). In addition to solubility, carbohydrate chain length also affects butyrate production (38). Generally, NDCs with longer chains have relatively lower utilization rate and are more resistant to intestinal fermentation, which results in a more distal type of metabolism (58). In contrast, NDCs with a shorter chain are more accessible to the microflora, which produces butyrate more rapidly (58, 59). Differences in the orientation and the position of the glycosidic bond and monomeric composition of NDCs may affect the production of butyrate (38).

The gut environment also impacts butyrate production. Particularly, the gut pH tremendously affects the concentration of butyrate because of differing tolerance to low pH of the major bacterial functional groups that comprise the human colonic microbiota (60). PH modulates microbial colonization in the upper gastrointestinal tract (61), and affects the metabolic activity and composition of microbial community (62). Some studies have shown that butyrate formation is affected by lowering of the gut pH (5.5) (60, 63, 64). Butyrate producers are also sensitive to iron availability, while butyrate production is enhanced at high iron concentrations (65, 66). Furthermore, the concentration of intestinal gases, like the oxygen and hydrogen also influences butyrate formation (67, 68).

### 2.4 Butyrate absorption

Butyrate is mainly considered be absorbed *via* active transport mediated by monocarboxylate transporters (MCTs) (69). MCT is coupled to a transmembrane H+-gradient that aids the transport and absorption of butyrate (70, 71). Specific isoforms of MCTs that are expressed in the colonic cell membrane that faces the lumen recognize butyrate as a substrate (72). However, the driving force for the uphill entry of butyrate from the lumen into colonocytes *via* MCTs is very little, because the magnitude of the transmembrane H+ gradient across the colonocyte apical membrane is puny (73). Out of the four functional MCTs, butyrate is mainly the substrate of transporters MCT1 (SLC16A1) and MCT4 (SLC16A3) (74). MCT1 is expressed both in the apical membrane and basolateral membrane of colonic epithelium whereas MCT4 specifically in the basolateral membrane (75). In terms of structure, human MCT1 consists of 500 amino acids with 12 putative transmembrane domains, with both amino- and carboxy-termini positioned on the membrane’s cytoplasmic side (76). MCT1 is highly expressed in Caco-2 cell and play a major role in the apical uptake of butyrate (77, 78). MCT4 consist of 465 amino acids and 12 transmembrane domains (79). MCT4 was shown to be a high-affinity butyrate transporter in gut epithelial cells (80). Beyond that, solute carrier (SLC) family 5 member 8 (SLC5A8), which is a Na+-coupled co-transporter and also known as sodium-coupled monocarboxylate transporter 1 (SMCT1), facilitates the transport and absorption of butyrate (81, 82). SMCT1 consists of 610 amino acids (83), primarily distributing in kidney and intestine (84). Butyrate is more readily transported by SLC5A8,which is relatively a high-affinity transporter with affinities for the butyrate in the sub-millimolar range (73). Some studies have also reported simple passive diffusion as a convincing model of butyrate transport (85).

### 2.5 Butyrate distribution

Up to 95% of butyrate produced by the gut microbiota is absorbed and used in colonocytes and only a very small part is absorbed into the circulation (26) *via* the hepatic portal vein, which connects the gastrointestinal tract, spleen and liver (86, 87). Butyrate concentrations in the portal vein are ~18µmol/l in a fasting human and 14-64µmol/l in sudden death victims (86, 88), while the concentrations in peripheral blood are low to ~20% of portal vein concentrations (89). Some studies have reported that oral delivery of dietary fiber or butyrate, or colonic infusion of butyrate was able to increase the butyrate concentration in the plasma of circulating blood (90–92). A study that followed the distribution of ^11^C-labeled butyric acid in baboons found relatively high accumulation of the label in the spleen, and pancreas (93). Another study reported distribution of ^13^C-labeled butyrate in the intestine, brain, brown adipose tissue (BAT), white adipose tissue (WAT), and especially the brain (94). Liu et al. found slightly elevated butyrate levels in the brains of mice supplemented with live *Clostridium butyricum* (95).

### 2.6 Butyrate metabolism and excretion

Butyrate is predominantly metabolized in the colon as an energy substrate, with small concentrations utilized by the liver and kidneys, providing up to 70% of the energy needs of colon cells, and the amount of butyrate metabolized was followed by the excretion of CO_2_ in breath (96, 97). In colon cells, butyrate is metabolized by mitochondrial β-oxidation to generate NADH, H+ and acetyl-CoA, and in turn can further be used to generate ATP in the citric acid cycle in the mitochondria (97). Butyrate enters into the tricarboxylic acid (TCA) cycle as acetyl-CoA and is converted to citrate, oxaloacetate, triosephosphate, and subsequently in glucose synthesis (98). In addition to the above, butyrate is metabolized to produce fatty acids, cholesterol, and ketone bodies (98). Approximately 10% of butyrate is excreted in the feces (41) and fecal butyrate levels are increased by a diet high in dietary fiber or resistance starches (99).

## 3 Cellular signaling pathways of butyrate

### 3.1 G protein-coupled receptors (GPCRs)

Butyrate is the ligand for metabolite-sensing G-protein coupled receptors (GPCRs), mainly GPR43, GPR41, and GPR109a (100), which are also known as free fatty acid receptors 2 (FFAR2), free fatty acid receptors 3 (FFAR3), and Hydroxy-carboxylic acid receptor 2(HCA2), respectively (101, 102). The expression of GRP41/FFAR3 receptors are mainly observed in peripheral nerves, enteroendocrine L and K cells, white adipocytes, pancreatic β- cells, thymus cells, and myeloid dendritic cells, and GRP43/FFAR2 receptors are expressed in white adipocytes, enteroendocrine L cells, intestinal epithelial cells, pancreatic β- cells, and several immune system cells (103). In colonic macrophages and dendritic cells, GPR109A signaling activates the inflammasome pathway, resulting in the differentiation of regulatory T cells and IL-10- producing T cells (104). FFAR2 and FFAR3 are activated by all three major SCFAs (105, 106), while butyrate is the only SCFA that can bind to HCA2 (104). In knockout GPR41 mice, the receptor was found to be involved in the release of peptide YY (PYY), intestinal transit rate, and energy harvesting from food (107). GPR43 knockout mice display weight gain, increased adiposity, and reduced systemic insulin sensitivity, while adipose tissue-specific GPR43 overexpression protects mice against the development of obesity (108). Butyrate directly regulates GPR41-mediated sympathetic nervous system activity and thereby controls body energy expenditure in maintaining metabolic homeostasis (109). These may be associated with the effect of butyrate on obesity.

### 3.2 Histone deacetylases (HDACs)

Butyrate is also a histone deacetylase inhibitor (HDACi). Anticancer activity of butyrate has been found to be mediated by HDAC inhibition, and includes inhibition of cell proliferation, induction of cell differentiation, apoptosis, and induction or repression of gene expression (110–112). Butyrate also down-regulates proinflammatory effectors by histone deacetylase inhibition to regulate intestinal macrophage function (113). A possible mechanism involves butyrate inhibition of the recruitment of HDACs to the promoter by the transcription factors specificity protein 1/specificity protein 3 (Sp1/Sp3), leading to histone hyperacetylation (112). In addition, studies have shown that, as a histone deacetylase inhibitor, butyrate promoted pancreatic β-cell differentiation, which was seen as having potential for the treatment of diabetes (114, 115).

## 4 Effect of butyrate on obesity

A growing number of studies have reported effects of butyrate on obesity, involved body weight, fat mass and obesity-related glucose and lipid metabolism (**Table 1**).

**Table 1:** Effect of butyrate on obesity, involved body weight, fat mass and obesity-related glucose and lipid metabolism.

Experimental models	Experimental design	Body weight and fat	Obesity-related glucose and lipid metabolism	Ref.
Male C57BL/6J mice with HFD	5% (w/w) sodium butyrate supplementation in HFD for 16 weeks. Pretreated with HFD for 16 weeks, and then administrated butyrate to obese mice for 5 weeks.	Butyrate prevented against body weight gain and fat content. Treatment with butyrate, body weight was reduced by 10.2%, and fat content was reduced by 10%.	Butyrate improved fasting glucose, insulin levels, homeostasis model assessment for insulin resistance (HOMA-IR) and insulin tolerance.	(116)
C57BL/6N male mice with HFD	5% (w/w) sodium butyrate supplementation in HFD for 4 weeks	Butyrate blocked HFD-induced weight gain.	Butyrate reduced fasting insulin levels, and improved oral glucose tolerance.	(117)
Male C57Bl/6J mice with HFD	5% (w/w) sodium butyrate supplementation in HFD for 12 weeks. Pretreated with HFD for 12 weeks, and then supplemented the HFD with butyrate for 6 weeks	Butyrate reduced in body weight, white adipose tissue (WAT) mass and adipose cell size, and prevented/treated HFD-induced obesity	Butyrate reduced fasting glucose and insulin levels, improved glucose tolerance and insulin sensitivity.	(118)
male C57BL/6J mice with HFD	5%(w/w) sodium butyrate supplementation in HFD for 8 weeks.	Butyrate attenuated HFD-induced increases in body fat, body weight, and adiposity	Butyrate improve glucose tolerance and insulin sensitivity.	(119)
C57BL/6 J male mice with HFD	5% (w/w) sodium butyrate supplementation in HFD for 16 weeks.	Butyrate significantly suppressed the HFD-induced body weight gain.	Butyrate attenuated the increases in glucose, insulin, triglycerides and cholesterol promoted by the HFD	(120)
Male C57BL/6JUnib mice with HFD	5% (w/w) sodium butyrate supplementation in HFD.	Butyrate resulted a significant decrease in body weight gain and 27.7% in adipose tissue accumulation.	Butyrate treatment blocked the development of insulin resistance and hyperinsulinemia states induced by HFD.	(121)
APOE*3-Leiden. CETP (E3L.CETP) mice with HFD.	5% (w/w) sodium butyrate supplementation in HFD for 9 weeks.	Butyrate decreased body weight and the weight of the gonadal (g) white adipose tissue (WAT) pad by −69%.	Butyrate significantly decreased plasma TG, fasting glucose, fasting insulin levels and homeostatic model assessment of insulin.	(122)
Female C57BL/6 mice with WSD	5% (w/w) sodium butyrate supplementation in WSD for 12 weeks	Butyrate significantly attenuated body weight gain, liver weight gain and hepatic lipid accumulation	Butyrate significantly reduced plasma triglyceride level	(34)
Male ApoE KO mice with HFD	1% butyrate (v/w, 10 mL/kg diet, using 10 mL of butyric acid adjusted with 4 N NaOH to pH 7.2.) supplemented in HFD for 10 weeks.	Butyrate reduces weight gain and adipocyte expansion in obese mice	Butyrate improved oral glucose tolerance, insulin sensitivity and serum adiponectin levels.	(123)
Male C57BL/6J mice with HFD	Sodium butyrate (400 mg/kg) supplemented in HFD for 16weeks	Butyrate supplementation alleviated weight gain.	Butyrate significantly improved HFD-induced glucose tolerance and insulin sensitivity.	(124)
C57BL/6J male mice with HFD	0.1 M sodium butyrate in the drinking water for 12 weeks	Butyrate significantly lowered the epididymal subcutaneous fat weight, body weight gain and lipid accumulation		(125)
Male C57Bl/6NTac mice with HFD	2.5 mM Sodium butyrate in 1 mL was orally administrated in drinking water using mouse catheters for five consecutive days in a week for the subsequent 6 weeks	Butyrate suppressed the HFD-induced weight gain.		(126)
Male specific pathogen-free (SPF) C57BL/6J mice	Pretreated with HFD for 8 weeks and then HFD-fed mice treated by gavage with 80mg sodium butyrate in 1 mL deionized water per mouse every other day for 10 days.	Butyrate treatment reduced the body weight, the epididymal fat mass and lipid deposition in the muscle.	Butyrate alleviated glucose tolerance, and restores plasma level of glucose and insulin.	(127)
Male LDLr-/- Leiden mice with HFD	One group, representing mid-adulthood, received a HFD at three months of age and received HFD enriched with 5% (w/w) sodium butyrate at seven months for two months. One group, representing late adulthood, received a HFD at six months of age and received HFD enriched with 5% (w/w) sodium butyrate at ten months for two months.	Butyrate in late adult mice on HFD resulted in a body weight loss of 23%. Butyrate intervention restored epididymal, inguinal and omental fat.	Butyrate lowered the plasma levels of cholesterol, triglycerides and insulin in both mid- and late adult mice.	(128)
Specific pathogen-free (SPF) C57BL/6J mice with HFD	Pretreated with HFD for 8 weeks and then HFD-fed mice treated by gavage with 80mg sodium butyrate in 1 mL deionized water per mouse every other day for 10 days.	Butyrate treatment significantly reduced body weight and epididymal fat mass, decreased the size of the fat deposits and adipose cells.		(129)
Male C57Bl/6 mice with HFD	Pretreated with HFD for 12 weeks and then HFD-fed mice treated by gavage with sodium butyrate (100 mg/kg/die, water as vehicle)	Butyrate treatment significantly reduced body weight and lipid accumulation.	Butyrate significantly reduced serum triglycerides, cholesterol, ALT and Lps, and improved glucose tolerance and insulin sensitivity.	(130)
Male LDLr-/-.Leiden mice with HFD	One group, representing mid-adulthood, received a HFD at three months of age and received HFD enriched with 5% (w/w) sodium butyrate at seven months for two months. One group, representing late adulthood, received a HFD at six months of age and received HFD enriched with 5% (w/w) sodium butyrate at ten months for two months.	Butyrate significantly reduced epididymal adipocyte size in both age cohorts. Butyrate intervention reduced inguinal adipocyte size in mid-adult mice but not in late-adult mice,	Epididymal and inguinal adipocyte size were positively associated with body weight, and cholesterol, triglyceride. Only in late adulthood, epididymal adipocyte size correlated positively with plasma insulin levels.	(131)
Male specific-pathogen-free Sprague Dawley rats with HFD	Pretreated with HFD for 9 weeks and then HFD-fed mice treated by gavage with sodium butyrate (300 mg/kg body weight) every other day for 7 weeks.	Butyrate treatment reduced the body weight, liver weight, and epididymal fat weight.		(132)
Male C57BL/6J mice with HFD	Pretreated with HFD for 8 weeks and then HFD-fed mice treated by gavage with sodium butyrate (200 mg/kg body weight) for 8 weeks.	Butyrate significantly reduced the body weight, hepatic steatosis and lipid accumulation.	Butyrate significantly reduced fasting serum levels of glucose, ALT and AST.	(133)
Male Specific-pathogen-free Sprague-Dawley rats with HFD	Pretreated with HFD for 9 weeks and then HFD-fed mice treated by gavage with sodium butyrate (300 mg/kg body weight) every other day for 7 weeks.	Butyrate treatment significantly reduced the body weight.	Butyrate treatment improved serum glucose level and glucose tolerance.	(134)
Male C57Bl/6J mice with HFD	Pretreated with HFD for 2 weeks, and then administered sodium butyrate(1000 mg/kg body weight) by daily oral gavage for 2 weeks	Butyrate reduced HFD-induced body weight gain and fat mass as well as scWAT and BAT weight.	Butyrate reduced serum fasting glucose and TG levels.	(135)
Specified pathogen-free (SPF) male C57BL/6 mice with HFD	Pretreated with HFD for 8 weeks and then HFD-fed mice treated by gavage with sodium butyrate(200 mg/kg body weight)for 8 weeks.	Butyrate attenuated HFD-induced weight gain	Butyrate improved fasting blood glucose, insulin sensitivity, and HOMA-IR.	(136)
Children with obesity who age 5 to 17 years and body mass index (BMI) greater than the 95th percentile for sex and age	The butyrate group received standard care for pediatric obesity plus sodium butyrate capsules, 20 mg/kg body weight per day, up to a maximum of 800 mg/d for 6 months.	children treated with butyrate had a higher rate of BMI decrease greater than or equal to 0.25 SD scores at 6 months (96% vs 56%, absolute benefit increase, 40%; 95% CI, 21% to 61%; P < 0.01).	waist circumference, −5.07 cm (95% CI, −7.68 to −2.46 cm; P <.001); insulin level, −5.41 μU/mL (95% CI, −10.49 to −0.34 μU/mL; P = .03); HOMA-IR, −1.14 (95% CI, −2.13 to −0.15;P = .02).	(137)

### 4.1 Body weight and fat mass

Most studies showed that dietary supplementation of a high-fat diet (HFD) with 5% (w/w) sodium butyrate significantly reduced body weight gain in mice compared with that induced by the HFD alone (34, 116–122), whatever a long-term supplementation for up to 16weeks (116, 120) or a short-term supplementation for down to 4 weeks (117). Although the dosage of addition of sodium butyrate supplementation varied, it was consistently effective in reducing body weight (123, 124), whether it was supplied in drinking water or administered by gavage (125, 126). Most of these studies also reported that butyrate supplementation can reduce fat mass, suppress adipose tissue accumulation and hepatic lipid accumulation (34, 116–119, 121–123, 125). Such evidence suggests that butyrate prevents diet-induced obesity (DIO) (34, 116–126).

Butyrate was also effective for treating diet-induced obesity (116, 118, 127–136). Studies that pretreated mice or rats with HFD for some time and then treated them by adding sodium butyrate dietary supplementation showed significant decreases in body weight and fat mass (118, 128, 131). In addition to dietary supplementation, more studies that treated HFD-fed mice or rats with oral delivery of sodium butyrate *via* gavage also significantly reduced body weight and fat mass (116, 127, 129, 130, 132–136). Not only that, an early study revealed that butyrate directly inhibited the proliferation of adipoblasts derived from lean and obese Zucker and WDF rats, and decreased the lipogenic capacity (138). Notably, a study showed that butyrate intervention did not significantly reduce bodyweight in mid-adult mice on HFD but resulted in a body weight loss in late-adult mice on the HFD (128). Another study showed that butyrate intervention reduced inguinal adipocyte size in mid-adult mice but not in late-adult mice (131). The results suggest that the effect of butyrate on obesity is associated with age (128, 131). Recently, a randomized clinical trial about pediatric obesity showed that butyrate decreased the BMI SD scores of obese children (137).

However, butyrate treatment failed to reduce body weight gain and fat mass increased in diabetes (139). It also showed that butyrate did not affect the body weight and fat mass of lean individuals fed a standard diet (121, 123). But in contrast, a study showed that dietary butyrate reduced body weight under standard diet conditions (35). Interestingly, some studies reported that maternal butyrate supplementation increased offspring body weight (140, 141). And the body weight of piglets which were orally gavaged with butyrate from day 4 after birth increased significantly compared with saline-treated control pigs (142). These results suggest that the effect of butyrate on individuals without diet-induced obesity is complex and uncertain.

### 4.2 Obesity-related glucose and lipid metabolism

Butyrate supplementation in HFD can prevent HFD-induced elevation of fasting glucose and insulin levels, and improve hyperglycemia and hyperinsulinism (116–118, 120–122). Butyrate supplementation can also improve glucose tolerance and insulin sensitivity (116–119, 121–124). Moreover, butyrate supplementation can reduce the serum triglycerides (TG) and cholesterol levels promoted by HFD (34, 122, 123). Butyrate treatment also had therapeutic benefits in disorders of glucose and lipid metabolism in obese individuals which were induced by HFD pretreatment (118, 127, 130, 131, 133–136). Butyrate improved the glucose homeostasis and peripheral insulin-resistance induced by diabetes or HFD without changes in body weight and fat mass (139, 143, 144), which indicates that there are other mechanisms underlying butyrate regulation on glucose homeostasis besides variation in body weight and body composition. It is worth mentioning that butyrate always improves the dyslipidemia under most conditions (34, 118, 122, 123, 127, 130, 131, 133–135, 139, 143), even in a randomized crossover trial which treats overweight/obese men with butyrate mixtures (91).

## 5 Mechanisms of butyrate effect for obesity

**Figure 1:**
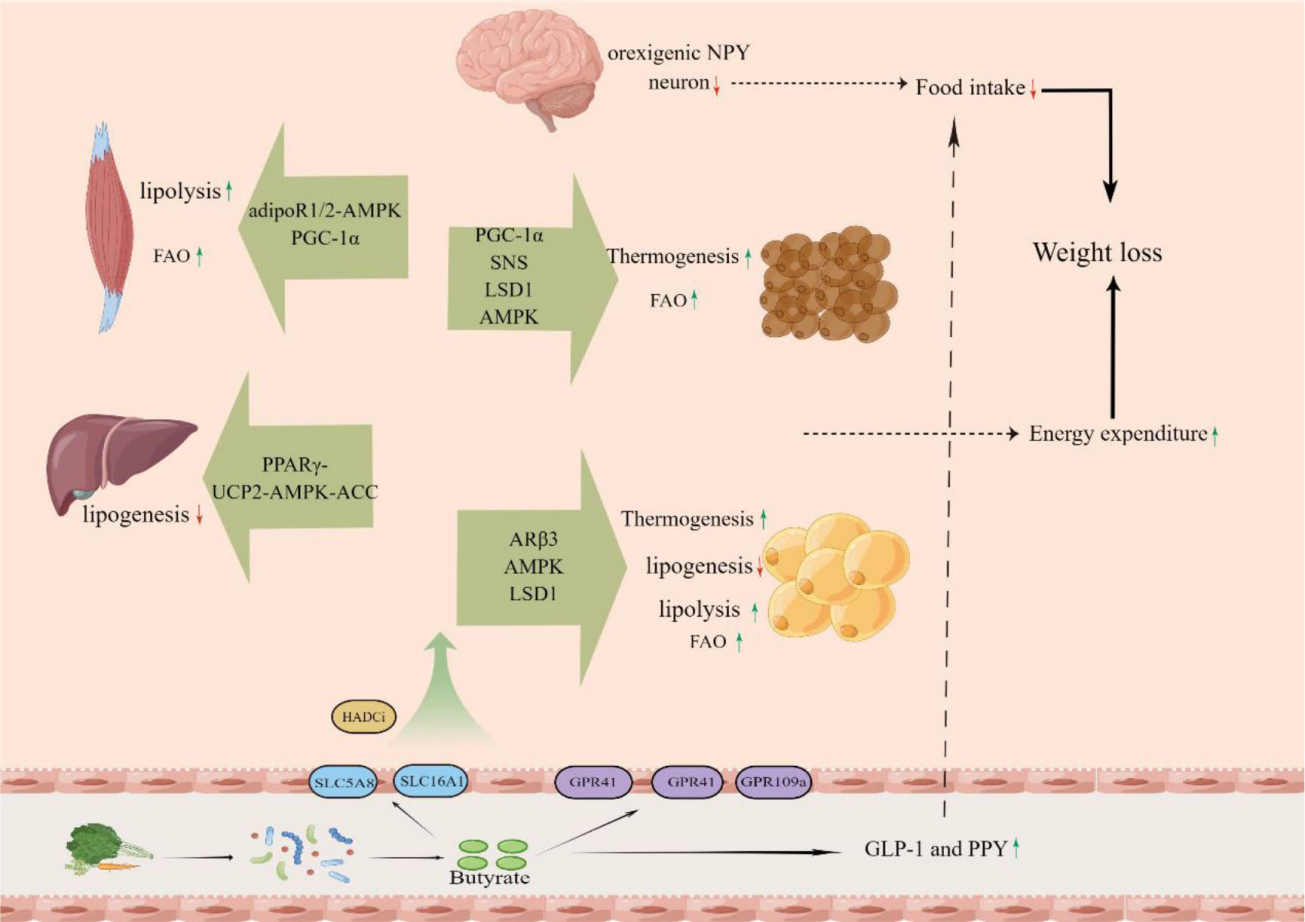
The butyrate, which is produced from dietary fiber by gut microbiota in the colon, is absorbed into the body and increase energy expenditure of muscle, liver, white fat and brown fat and decrease energy intake.

The root cause of obesity is that energy intake exceeds energy expenditure (14, 145). Accordingly, the central to obesity treatment are the increase of energy expenditure and the decrease of energy intake (146, 147). Butyrate plays an important role in both energy expenditure and energy intake through a variety of mechanisms (**Figure 1**).

### 5.1 Thermogenesis of adipose tissue

In some studies, butyrate stimulated thermogenesis of brown adipose tissue (BAT) *via* the upregulation of uncoupling protein-1 (UCP1) expression, thus increasing energy expenditure and improve HFD-induced obesity (116, 122, 135). BAT, whose brown adipocytes are packed with mitochondria that contain uncoupling protein-1 (UCP1), is a key site of thermogenesis and energy expenditure (148). UCP1, a very important regulatory factor in thermogenesis, which residing in the inner mitochondrial membrane, uncouples mitochondrial respiration from ATP synthesis resulting in thermogenesis (148). It is a significant mechanism for butyrate to promote weight loss by increasing energy expenditure that BAT dissipate chemical energy in the form of heat *via* UCP1 to regulate body temperature and whole body energy expenditure.

Further studies did find that butyrate can increase the expression of peroxisome proliferator–activated receptor (PPAR)-γ coactivator-1α(PGC-1α) (116), which is a critical regulator of mitochondrial function, and stimulates thermogenesis *via* the upregulation of UCP1 expression (149). As increased expression of PGC-1α in BAT was reported in a study of the effects of butyrate supplementation, and was positively associated with GPR43 (120). This evidence may suggest that butyrate promote thermogenesis of BAT in partly through activating GPR43 to upregulate PGC-1α. In addition, it has also shown that butyrate stimulated PGC-1α activity by activating AMP-activated protein kinase (AMPK) and inhibiting histone deacetylases (116).

Another convincing mechanism is that butyrate improves BAT thermogenic capacity by increasing sympathetic outflow towards BAT, as butyrate was found to increase the protein expression of tyrosine hydroxylase (TH) (122), a marker of sympathetic nerve activity (150). Furthermore, butyrate treatment did not influence the UCP-1 expression in BAT of vagotomised mice (122), which indicates that the sympathetic nervous system was necessary for butyrate-induced BAT activation.

Recent research identified LSD1, which is an important factor in the regulation of BAT thermogenesis function (151), as a potential mediator of butyrate-induced thermogenesis in BAT, because it can be activated by butyrate to increase UCP1 expression (135). In this study, we can see that LSD1 knockout blocked the butyrate-induced increase in thermogenesis and energy expenditure in BAT (135). The results of experiments with adipocytes also showed that the effects of butyrate on LSD1 and UCP1 were in an AMPK-independent manner (135). These results suggest that butyrate, taken up and metabolized *via* MCT1 and ACSM3, can directly activate LSD1 to increase the UCP1 expression to mediate thermogenesis in BAT.

Butyrate can change the composition of gut microbiota and improve the disturbed intestinal flora of obese mice (124, 125). Some researches have shown that microbiota depletion impaired thermogenesis of BAT and that butyrate supplementation partially rescued thermogenesis function (94, 135). Microbiota depletion by different cocktails of antibiotics (ABX) or in germfree (GF) mice was reported to impaired the thermogenic capacity of BAT by decreasing the expression of UCP1 (94). It is possibility that butyrate enhance the function of brown fat by regulating the intestinal microflora of obese individuals.

Beige adipocytes that appear in WAT are similar to brown adipocytes in that they release energy as heat and the thermogenesis is mediated by UCP1 (152). Butyrate supplementation can increase the expression of UCP-1 to mediate thermogenesis in WAT and beige adipocyte markers (Tbx1, Tmem26, CD137) (120, 135). GPR43, GPR41 and LSD1 are needed in WAT for butyrate regulation on beiging (120, 135).

### 5.2 Lipogenesis

It is reported that butyrate can reduce the body weight and fat mass by decreasing lipogenesis in liver and adipose tissue (118, 133). Butyrate supplementation decreased Fatty acid synthase (FAS) expression to reduce lipid synthesis, especially triglycerides (118). This may be attributed to the effect of butyrate in activating AMPK to induce phosphorylation of its downstream target acetyl-CoA carboxylase (ACC) (118, 133). Further, increase of the AMP-to-ATP ratios, which is a direct activator of AMPK, may have been caused by proton leak *via* UCP2 in butyrate-feed animals, and the disruption of the activity of PPARγ abolished the SCFA-induced increase of the UCP2-pAMPK-pACC pathway activity (118). These results suggested that butyrate activated the UCP2-AMPK-ACC pathway by downregulating the peroxisome proliferator–activated receptor-γ (PPARγ), which can reduce the lipogenesis (118). Other studies suggest that butyrate can inhibit lipid synthesis by inducing the p-AMPK/p-ACC pathway through upregulation of hepatic Glucagon-likepeptide-1 receptor(GLP-1R) (133, 153).

### 5.3 Adipose lipolysis

Adipose tissue, especially the white adipose tissue, is the largest energy reservoir of body, and has a critical role in the regulation of energy homeostasis (154). Thus, increasing fat mobilization in adipose tissue is an effective strategy to control or treat obesity. A study suggested that butyrate stimulated adipose lipolysis by the phosphorylation of adipose triglyceride lipase (ATGL) and hormone-sensitive lipase (HSL), which are the two major lipases for fat mobilization from triglyceride stores in adipose tissue, and then significantly reduced the epididymal fat mass and body weight (129). Some studies also found that butyrate supplementation reversed the reduction of adipose HSL and lipoprotein lipase (LPL) in obese individuals, causing an increase in the fat hydrolyzed (120). The decrease of lipid content, associated with a significant up-regulation of HSL and LPL was also found in muscle (127).

Butyrate modified histone acetylation on the promoter of beta3-adrenergic receptors (ARβ3) gene, and increased the activation of its downstream signaling molecule cAMP-dependent protein kinase (protein kinase A, PKA) (129). Activating ARβ3, which also belongs to the family of G protein-coupled receptors and is widely expressed in adipose tissues to play important roles in lipolysis (155), stimulates lipolysis by activating phosphorylation of HSL and ATGL (156, 157). HSL and ATGL phosphorylation depend on PKA activation (158, 159). The results suggest that butyrate stimulates adipose lipolysis through histone hyperacetylation-associated AR3β activation in WAT. In muscle, reduced muscle lipid content and up-regulation of Hsl and Lpl mRNA expression are due to butyrate which up-regulates the expression of adiponectin receptors as an HDAC inhibitor (127).

### 5.4 Fatty acid oxidation

Increasing fatty acid oxidation (FAO) is able to reduce fat accumulation and improves obesity (160). The increase in energy expenditure increases FAO because lipids and fatty acid are the main energy substrates for cellular energy expenditure. Some studies showed that butyrate treatment reduced the Respiratory exchange ratio(RER), suggesting an increase in FAO in response to butyrate (116, 118, 135). FAO is associated with expression of carnitine palmitoyltransferase-1 (CPT-1), which includes three isoforms: CPT-1A(liver), CPT-1B (muscle and heart), and CPT-1C (brain) (160, 161). The increases of CPT-1 induced by butyrate are found in muscle, liver and adipose tissue (116, 118, 120, 135). Butyrate treatment significantly reduced muscle content of TG and total cholesterol compared with mice fed a HFD (127). In addition to CPT-1, butyrate also increased the expression of COX-1(cytochrome c oxidase), UCP2 and UCP3, which can facilitate FAO in skeletal muscle (162). Butyrate induced the transformation of skeletal muscle fiber from the glycolytic muscle fiber type to the oxidative type (116), which is rich in mitochondria, red in color, and active in fat oxidation for ATP biosynthesis (163). The increase of FAO induced by butyrate in skeletal muscle may ascribe to the increased expression of PGC-1α (116). Activation of AMPK and inhibition of HDAC may contribute to the PGC-1α regulation (116). As a HDAC inhibitor, butyrate can activate AMPK by enhancing the expression of adiponectin receptors (adipoR1/2) (127). The increase of the FAO induced by butyrate in BAT may attribute to the energy demands of thermogenesis (135). Butyrate can reduce HFD-induced body weight gain and fat mass by enhancing energy expenditure through increased lipid oxidation in WAT (118, 120). The effect in WAT is due to activation of the UCP2-AMPK-ACC pathway by butyrate depends on PPARγ (118). Butyrate has also been shown to increase lipid oxidation by directly activating the AMPK/ACC pathway to reduce fat mass (130).

### 5.5 Mitochondrial function

A study showed that butyrate reduced lipid accumulation by regulating liver mitochondrial function, reducing liver mitochondrial energy efficiency, and improving the capability of mitochondria to utilize fat as metabolic fuel (130). Butyrate can stimulate mitochondrial oxidative phosphorylation in WAT through histone hyperacetylation-associated ARB3 activation (129). Short-term oral administration of butyrate can alleviate diet-induced obesity in mice by stimulating mitochondrial function in skeletal muscle (127). Butyrate has also been reported to increase the number of mitochondria in skeletal muscle (116).

### 5.6 Appetite and intake

Studies have shown that butyrate suppressed food intake, which contributed to reduce body weight and fat mass (117, 122). Butyrate administration *via* intragastric gavage but not intravenous injection significantly reduced acute food intake within 1 hour after refeeding and cumulative food intake over 24 hours (122). Others found that butyrate did not influence fat absorption by the gastrointestinal tract (116). But some studies showed that butyrate did not reduce the food intake (116, 118), and butyrate has unpleasant taste. This is still a controversial issue that butyrate can increase satiety and decrease appetite.

The appetite is mainly affected by the neural circuits and gut hormones (164, 165). A study showed that butyrate reduced the activity of orexigenic neuropeptide Y (NPY) neuron in the hypothalamus and neuron activity within the nucleus tractus solitarius (NTS) and dorsal vagal complex (DVC) in the brainstem (122). Not only that, butyrate-induced satiety and decreasing food intake was completely abolished by subdiaphragmatic vagotomy, which indicated that the gut-brain neural circuit is necessary for butyrate-induced satiety (122). Butyrate can also significantly increase the levels of gut hormones in the colon and plasma such as GLP-1 and PPY, which can reduce the appetite and food intake (91, 117). Evidence indicated that the effect of butyrate in inhibiting weight gain and food intake in Ffar3 knockouts was to the same extent as in wild-type mice, but stimulation of PYY and GLP-1 by butyrate was blunted in the absence of FFAR3 (117). The peptide hormone leptin can also regulate food intake and body mass (166). Butyrate can increase the leptin production in DIO mice (117, 123, 127, 130, 131).

## 6 Obesity-induced complication

Obesity or overweight is an important determinant of a range of health problems and increases the risk of many related diseases including type 2 diabetes mellitus (T2DM), CVD, nonalcoholic fatty liver disease (NAFLD), impaired cognition, and others (7, 167–169). Butyrate has a role in control of obesity-induced complications not only by its effect on weight loss but also because of many other mechanisms of action.

### 6.1 Type 2 diabetes mellitus

The epidemic of diabetes mellitus, which is the ninth major cause of death, poses a major global health threat and about 1 in 10 adults worldwide now has type 2 diabetes mellitus (170). Obesity is one of the strongest risk factors and predisposition for type 2 diabetes (169, 171). Gut microbiota play a key role in obesity and diabetes (172). As a main metabolic product of intestinal microbiota, butyrate can improve HFD-induced glucose homeostasis and insulin resistance, which are directly associated with development of diabetes mellitus (116–118, 121, 124, 127, 130). Butyrate activated the hormone signaling such as protein kinase B (PKB/Akt), and increase the expression of glucose transporter (Glut4) (116, 123, 124, 130).

Evidence showed that butyrate can reduce HFD-induced pancreatic beta cell dysfunctions (121, 143), which has the beneficial effect on glucose homeostasis and suppresses the development of diabetes (173). Butyrate can improve pancreatic β cell development, proliferation, and function *via* the inhibition of HDACs (174, 175), protect pancreatic Beta cells from Cytokine-Induced Dysfunction (176), increase the pancreatic Beta cells viability and prevent pancreatic Beta cell-death during exposure to streptozotocin (177). Increased oxidative stress is one of the important factors which can lead insulin resistance and contribute to the development of T2DM (178). A study showed that butyrate stimulated transcription of downstream antioxidant enzymes *via* the activation of nuclear factor E2-related factor 2, thus contributing to the amelioration of HFD-induced oxidative stress and insulin resistance (134). Butyrate directly induced intestinal gluconeogenesis *via* upregulating key enzymes G6PC and PCK1 with a cAMP-dependent mechanism, which can activate hypothalamic nuclei to decrease liver glucose production and regulate insulin sensitivity and glucose homeostasis (35).

### 6.2 Nonalcoholic fatty liver disease

Over the past four decades, non-alcoholic fatty liver disease has become the most common chronic liver disorder, which has a global prevalence of 25% and is a leading cause of cirrhosis and hepatocellular carcinoma (179). Obesity is closely associated with the rising prevalence and severity of NAFLD (180). Some studies showed that butyrate improved the accumulation of fat in the liver, hepatic steatosis and inflammation, the liver index and serum levels of alanine transaminase (ALT) and aspartate transaminase (AST) induced by diet-induced obesity (34, 118, 121, 125, 132, 133, 144).

Several findings suggested that butyrate alleviates HFD-induced NAFLD by improving mitochondrial function and stimulating fatty acid β oxidation in the liver (118, 132, 181). Moreover, butyrate can inhibit the NF-κB signaling and NLRP3 inflammasome activation by up-regulating hepatic expression of peroxisome proliferator-activated receptor α (PPARα), which contribute to the alleviation of HFD-induced, NAFLD-associated hepatic inflammation (132, 144). Butyrate can protect against high-fat diet-induced oxidative stress in rat liver by promoting expression of nuclear factor E2-related factor 2 (134). Butyrate can decrease the lipopolysaccharide (LPS) and its receptor Toll-like receptor 4 (TLR4) in the liver by repairing HFD-induced damage to the intestinal mucosa and strengthened intestinal tight junctions, which is beneficial for the treatment of NAFLD (34, 136). Evidence showed that improved HFD-induced non-alcoholic steatohepatitis resulted from up-regulation of hepatic GLP-1R expression (133). Meanwhile, a recent study reported that butyrate protected mice against diet-induced NASH and liver fibrosis development by direct inhibition of collagen synthesis in hepatic stellate cells involving suppression of specific non-canonical TGF-β signaling pathways Rho-like GTPases and PI3K/AKT, and other important pro-fibrotic regulators (29). Butyrate also ameliorated NAFLD by upregulating miR-150 to suppress C-X-C Motif Chemokine Receptor 4 expression (182).

### 6.3 Neuropsychiatric dysfunction

Obesity is associated with an increased risk of neuropsychiatric disorders, including mood disorders, schizophrenia, major neurocognitive disorder or cognitive impairment, and neurodegenerative diseases (NDDs) (183–185). Evidence showed that Butyrate intervention restored HFD-induced spatial memory impairment, brain function, and neuroinflammation within the thalamus, cortex and hippocampus (128). Butyrate can also reverse HFD-induced social deficits and anxiety-like behaviors by regulating microglial homeostasis and reducing dendritic spine density in the bilateral medial prefrontal cortex (mPFC) (186). Analysis of the gut microbiome suggests that these beneficial effects may correlate with gut microbiota composition (128, 186). Butyrate can protect against NDDs by suppressing neurotoxicity and cell death. A study showed that butyrate attenuated the expression of P-53 and the neuroinflammation in the brains of HFD-fed mice (126). Butyrate also upregulated PPARγ/CREB, BDNF, and modulated the Nrf-2/HO-1 pathway in HFD mice brains, which play important roles in neuroprotective effect (126).

## 7 Conclusion and prospect

There is a lot of evidence supporting that butyrate, as a key mediator of microbiota in host metabolic control, has beneficial effects on obesity, especially weight loss. However, the role of butyrate is still controversial and not clear. First, as we mentioned earlier, the amount of butyrate in stools has been found to be higher in overweight and obese volunteers than in lean volunteers. These contradictory results led some researchers to believe that butyrate may contribute to the obesogenic phenotype. Besides, the range of action of butyrate is a controversial. Because there were many contradictory results about the effect of butyrate on weight loss in diabetic and non-obese individuals. Last but not least, the exact mechanisms of butyrate regulation need to be found out. For example, some studies reported that butyrate didn’t affect food intake, but some reported butyrate reduced appetite. Thus, we’ve done a lot of work on existing mechanisms of butyrate. These issues require largely meticulous and comprehensive studies to fully understand the role of butyrate in host metabolic health. More human clinical studies are needed to prove the effectiveness and specific effect of butyrate on obesity, and more diversified experimental models are needed to determine the effective conditions of butyrate in obesity. Finally, we need more persuasive experimental designs and studies to figure out the exact mechanism by which butyrate acts. Besides, the side effects of butyrate, like nausea and its unpleasant smell, also needed to be considered. It’s important to discover and try to remove the side effect of butyrate. To provide better strategies for obesity, further research based on published studies is needed in the future. However, experimental designs in rodent models may not be transferrable to human situations. The limited available human data are derived from studies with a relatively small sample size and short intervention period and didn’t show encouraging results. Therefore, it is needed to consider whether further research is worthwhile. We just hope that our work can contribute to related research and help someone who are interested in this field.

## Author contributions

Select the topic, YH and XY; literature collection, HT and WZ; writing-original draft preparation, KP; investigation and editing, WD and TL. All authors contributed to the article and approved the submitted version.
